# Radar Transformer: An Object Classification Network Based on 4D MMW Imaging Radar

**DOI:** 10.3390/s21113854

**Published:** 2021-06-02

**Authors:** Jie Bai, Lianqing Zheng, Sen Li, Bin Tan, Sihan Chen, Libo Huang

**Affiliations:** Institute of Intelligent Vehicles, School of Automotive Studies, Tongji University, Shanghai 201804, China; baijie@tongji.edu.cn (J.B.); zhenglianqing@tongji.edu.cn (L.Z.); lisen@tongji.edu.cn (S.L.); tanbin@tongji.edu.cn (B.T.); sihan.chen@tongji.edu.cn (S.C.)

**Keywords:** object classification, self-attention, MMW imaging radar, deep learning, autonomous driving

## Abstract

Automotive millimeter-wave (MMW) radar is essential in autonomous vehicles due to its robustness in all weather conditions. Traditional commercial automotive radars are limited by their resolution, which makes the object classification task difficult. Thus, the concept of a new generation of four-dimensional (4D) imaging radar was proposed. It has high azimuth and elevation resolution and contains Doppler information to produce a high-quality point cloud. In this paper, we propose an object classification network named Radar Transformer. The algorithm takes the attention mechanism as the core and adopts the combination of vector attention and scalar attention to make full use of the spatial information, Doppler information, and reflection intensity information of the radar point cloud to realize the deep fusion of local attention features and global attention features. We generated an imaging radar classification dataset and completed manual annotation. The experimental results show that our proposed method achieved an overall classification accuracy of 94.9%, which is more suitable for processing radar point clouds than the popular deep learning frameworks and shows promising performance.

## 1. Introduction

In recent years, autonomous driving technology [1] has developed rapidly and received wide attention. Autonomous vehicles mainly consist of several modules such as environment perception, path planning and decision control [2]. Among them, environment perception is significant and its good performance directly affects the downstream modules. The mainstream sensors in the environmental sensing module mainly consist of cameras, LIDAR and MMW radar [3]. It is indisputable that cameras and LIDAR fail to varying degrees in the rain, snow and fog and under operating conditions such as bright light and darkness, while the MMW radar is indispensable as it shows strong robustness under bad conditions [4]. The traditional MMW radar for commercial vehicles is affected by its resolution, making it challenging to perform object classification tasks [5]. Furthermore, it lacks object height information and serves only as a last line of defense in most autonomous driving systems, acting as an advanced warning. With the advent of a new generation of 4D high-resolution imaging radars [5,6,7], promising applications have been seen. The imaging radar can produce LIDAR-like point-cloud data, contain rich Doppler information and it has all-weather characteristics. However, research on related algorithms is still in the initial stage.

In terms of imaging radar hardware, Li et al. [6] proposed a novel 4D radar that operates at 79 GHz with 1.6 GHz bandwidth and uses frequency-modulated continuous wave (FMCW). The multiple-input multiple-output (MIMO) technique and binary phase shift keying (BPSK) coding were used for transmitting signals to obtain elevation information. Lastly, road edge height estimation, drain detection and parking lot detection were accomplished using this radar. Martin et al. [7] used a new antenna array device that provides the ability to measure angles in azimuth and elevation. In order to estimate the direction of arrival, examples were executed by combining them. In a related task based on imaging radar, Feng et al. [8] used a high-resolution MMW radar sensor to obtain a radar point-cloud representation for traffic surveillance scenes. Based on a new feature vector, it used a multivariate Gaussian mixture model (GMM) for radar point cloud segmentation in an unsupervised learning environment, i.e., “point-by-point” classification. Ibrahim et al. [9] used 3D point clouds generated by a planar phased array FMCW radar to detect different human motions. They extracted point clouds by calculating the direction of arrival of scattered points on the human body and used convolutional neural networks for classification with a final accuracy of 80%. Michael et al. [10] proposed a radar-centric autonomous vehicle dataset for 3D target detection based on radar, LiDAR and camera data. The dataset uses a Astyx 6455 HiRes [5] high-resolution imaging radar, which provides semi-automatically generated and manually refined 3D ground truth for object detection, with most of the objects being cars. Object classification datasets based on imaging radars are not yet available to the best of our knowledge; thus, object classification algorithms based on imaging radars need to be studied urgently.

In recent years, with the development of deep learning and artificial intelligence, deep neural networks have made impressive achievements. They are widely used in various fields [11], including data structures like point clouds. Since point clouds have characteristics such as permutation and orientation invariance [12], traditional convolutional neural networks are not suitable for handling such irregularly structured data. Hence, new strategies are needed to solve them. The first approach is based on multiple 2D views. MVCNN [13] performs a multi-view projection of the point cloud and conducts a convolution operation for each view by a view pooling procedure to aggregate the features of 12 views. Through this view pooling procedure, the features of 12 views can be aggregated. 3DMV [14] integrates RGB features and geometric features through a 2D-3D network. RotationNet [15] uses viewpoints of training images as potential objects for unsupervised learning of object poses. The second is the voxel-based approach. 3Dshapenet [16] uses a probability distribution of binary variables to represent a 3D voxel grid geometry and then uses a deep convolutional confidence network to process the 3D voxel data. VoxNet [17] uses probabilistic estimation to estimate the occupancy in 3D grids and uses three-dimension convolutional neural networks (3DCNN) to process the occupied grids to achieve object recognition. To solve the memory and computing costs caused by a large number of unoccupied voxels, OctNet [18] stratifies the input data into octrees according to the density of the input data. Moreover, graph convolution is also applied in point-cloud deep learning [19,20], such as in DGCNN [20], which uses EdgeConv [20] to extract local geometric information of local neighborhood graphs. With the advent of PointNet [12], point-wise networks began to appear. PointNet [12] uses multilayer perceptron (MLP) and max-pooling to ensure the permutation invariance of points, whose T-Net [12] structure ensures the rotation invariance. The whole network learns the point-wise features. PointNet++ [21] introduces a hierarchical neural network that recursively applies PointNet to nested partitions of a point set, thereby enhancing the learning of locally fine-grained features. Kd-networks [22] create an order of input points by using kd-trees so as to hierarchically extract features from leaf to root. PointConv [23] considers the weight as a continuous function of 3D coordinates, through which the convolution problem on 3D point clouds can be solved.

However, the object point cloud output by MMW imaging radar does not have clear shape characteristics like ModelNet40 [16] and Shapenet Parts Dataset [24], which have greater sparsity compared with the points collected by laser sensor or the points sampled by computer-aided design (CAD) maps. However, the reflection points from the object are of high quality and have Doppler velocity information, which is essential for the representation of the object. Multi-view projection and voxel-based methods generate a lot of invalid convolution calculations for such sparse point clouds, while point-wise methods consider more geometric shape information of the object and do not extract high-quality MMW point-cloud deep feature information well.

Recently, transformer [25] has dominated in natural language processing (NLP), as seen in its extensions BERT [26], transformer-XL [27], BioBERT [28], etc. Related research on transformer has been extended to the field of computer vision [29,30] and has achieved better results compared with traditional convolutional neural networks. The core of transformer is the self-attention module, which takes as input the sums of input embedding and positional encoding and maps them to produce query, key and value matrices for each word. The attention weights between any words can be generated by dot-product query and key matrices. The weighted sum of value and attention is the attention feature. This mechanism is actually well suited to dealing with data like point clouds. PCT [31] enhances input embedding by supporting farthest point sampling and nearest neighbor search. It applies transformer to point clouds and achieves good results.

Inspired by the transformer and self-attention [32] mechanisms, we propose Radar Transformer, an object classification network based on an MMW imaging radar. Using the self-attention mechanism, the local features and global features of the input radar point cloud are deeply fused at multiple levels. Combined with the two self-attention mechanisms, the features of the imaging radar point cloud can be better extracted. The experimental results show that the proposed method achieved the highest classification accuracy.

Our contributions are summarized as follows:

1. We generated an MMW imaging radar classification dataset. To the best of our knowledge, no publicly available classification dataset for high-resolution imaging radars has yet appeared. We collected dynamic and static road participants, including persons, cyclists, motorcyclists, cars and buses, and we manually annotated them. A total of 10,000 frames of data are available, with each object point containing XYZ spatial information, as well as Doppler velocity information V and signal-to-noise ratio (SNR).
2. We propose a new network architecture for imaging radar point-cloud classification based on transformer, which takes the five-dimensional information of radar point clouds as input. After the input embedding, the features are extracted by local hierarchical feature extraction and global feature extraction, with the two not being independent but exhibiting multilevel deep fusion. Combined with scalar attention and vector attention, deep features can be fully extracted.
3. Experiments show that our proposed network can better represent the imaging radar point cloud than the mainstream point-cloud network and exhibits state-of-the-art (SOTA) performance in the object classification task.

The remainder of this paper is organized as follows: Section 2 describes our network framework and the components of each module. Section 3 conducts experiments and evaluates the experimental results. Section 4 is the discussion. Lastly, in Section 5, we give conclusions and some future research.

## 2. Methodology

### 2.1. Network Architecture

**Figure 1:**
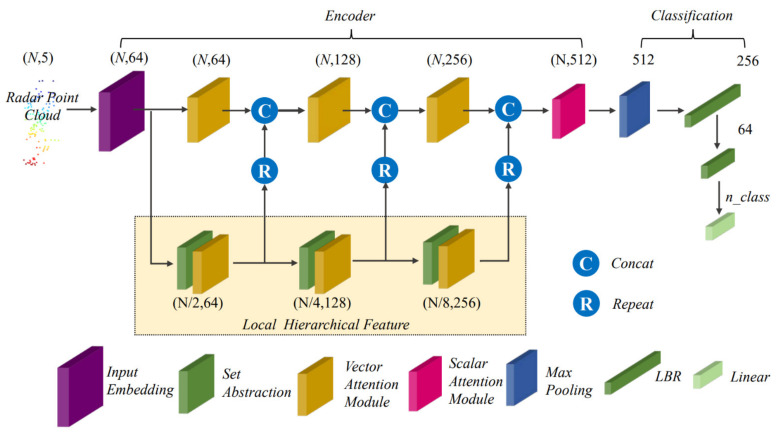
Radar Transformer architecture. The encoder mainly consists of an input embedding module, set abstraction module, vector attention module and scalar attention module, where the local hierarchical features are deeply fused with global features. The decoder consists of multiple linear layers. LBR combines linear, BatchNorm and ReLU layers. The numbers represent the feature dimensions after this operation.

The overall structure of the network is shown in Figure 1, and the specific structures of each module are described below.

In the feature encoder part, the input radar point cloud is first mapped into a feature vector in high-dimensional space by the input embedding. Inspired by MV3D [33], we propose a network structure with the deep fusion of global features and local hierarchical features. The input feature vector is divided into two branches. One of its branches goes through three stacked set abstractions and vector attention modules to gradually extract deep local features. The local features of each hierarchy are connected with the global features of the hierarchy as new global features to obtain the deeper feature representation of the input radar point cloud. The scalar attention module integrates the final obtained global features to obtain the final feature representation of the radar point cloud. In the feature decoder part, we feed the final feature representation into the MLP after max pooling to complete the classification task. The entire network deeply fuses local attention features with global attention features to obtain a better abstract representation of the radar point cloud.

Formally, given an input radar point cloud ${\left\{x_{i}\right\}}_{i=1,2,\ldots N}\in {\mathbb{R}}^{N\times d}$, which contains N points, each with d-dimensional features, the input point cloud is firstly transformed into a $d_{i}$-dimensional embedding feature $F_{i}\in {\mathbb{R}}^{N\times d_{i}}$ by input embedding. Then, the embedding features are then fed into the lower branches of the network, i.e., the three stacked set abstraction modules and vector attention modules used to extract deep local features. The local features at each hierarchy can be represented as

(1) $$\left\{\begin{array}{l} L_{1}{=\mathrm{VA}}_{L1}(SET_{1}(F_{i}))\\ L_{2}{=\mathrm{VA}}_{L2}(SET_{2}(L_{1}))\\ L_{3}{=\mathrm{VA}}_{L3}(SET_{1}(L_{2})) \end{array}\right.,$$

where ${\mathrm{VA}}_{L1}$, ${\mathrm{VA}}_{L2}$ and ${\mathrm{VA}}_{L3}$ denote the vector attention module of each hierarchy of the lower branch, $SET_{1}$, $SET_{2}$ and $SET_{3}$ denote the set abstraction operation of each hierarchy, and $L_{1}$, $L_{2}$ and $L_{3}$ are the local abstraction features of each hierarchy.

The main branch of the network gradually fuses global features with local features for a high-level abstract representation, and the global features at each hierarchical level can be represented as

(2) $$\left\{\begin{array}{l} G_{1}=\mathrm{concat}(VA_{G1}(F_{i}),R_{1}(L_{1}))\\ G_{2}=\mathrm{concat}(VA_{G2}(G_{1}),R_{2}(L_{2}))\\ G_{3}=\mathrm{concat}(VA_{G3}(G_{2}),R_{3}(L_{3})) \end{array}\right.,$$

where $VA_{G1}$, $VA_{G2}$ and $VA_{G3}$ are the vector attention modules in the main branch of the network, $R_{1}$, $R_{2}$ and $R_{3}$ are the repeat operations, and $G_{1}$, $G_{2}$ and $G_{3}$ are the global features of each hierarchical level, respectively.

Lastly, the obtained global features are integrated by the scalar attention module to get the final radar point cloud feature representation $F_{O}\in {\mathbb{R}}^{N\times d_{o}}$. After max pooling, it is fed into two LBRs (linear, BatchNorm, ReLU) and a linear layer to complete the probability prediction. The class label is the class with the highest score.

### 2.2. Input Embedding

In the field of NLP, embedding encodes words and maps the input words to a feature space to reduce the dimension of the input data and facilitate the calculation of the distance between words, such as word2vec [34]. In this paper, we simply encode the original input by the MLP consisting of two linear layers, i.e., the input radar point cloud ${\left\{x_{i}\right\}}_{i=1,2,\ldots N}\in {\mathbb{R}}^{N\times d}$ and the embedding output is $F_{i}\in {\mathbb{R}}^{N\times d_{i}}$. The input dimension d of the radar point cloud in our network is 5, which represents the data after normalization and augmentation (introduced in the experiment). In contrast to transformer, we do not include position encoding because we consider that the input radar points contain x,y,z,v,s, which represent the position in Euclidean space. For computational efficiency, we chose the dimension of each point to be $d_{i}=64$ after input embedding.

### 2.3. Set Abstraction

**Figure 2:**
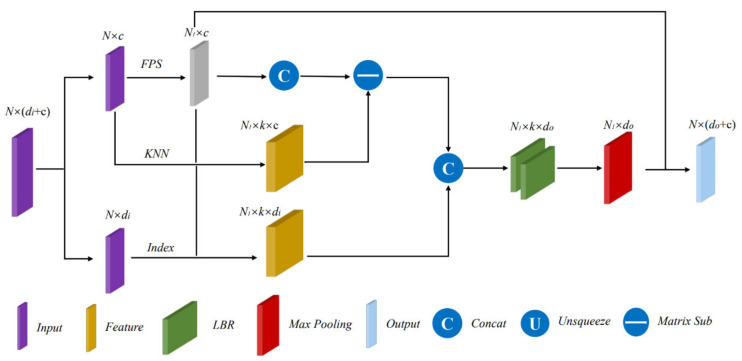
The structure of set abstraction.

Inspired by PointNet++, we adopted a hierarchical structure for set abstraction to extract deep local aggregation information gradually, and the structure of set abstraction is shown in Figure 2.

We input a matrix of dimension $N\times (d_{i}+c)$, where $d_{i}$ is the dimension of feature information and c is the dimension of spatial information. Unlike PointNet++, our spatial information consists of x,y,z,v,s representing the coordinates in the Euclidean space of dimension 5. Then, we perform farthest point sampling (FPS) [21] on the input points to obtain each local centroid point, which can better cover the point set in the Euclidean space. Thus, we get $N_{1}$ centroid coordinates $N_{1}\times c$. The FPS algorithm is shown in Algorithm 1. We use the first point in the input point set as the starting point. The distance between any two points $p_{i}$ and $p_{j}$ is expressed as follows:

(3) $$d(p_{i},p_{j})=\sqrt{{\left(x_{i}-x_{j}\right)}^{2}+{\left(y_{i}-y_{j}\right)}^{2}+{\left(z_{i}-z_{j}\right)}^{2}+{\left(v_{i}-v_{j}\right)}^{2}+{\left(s_{i}-s_{j}\right)}^{2}}.$$

**Algorithm 1** Farthest Point Sampling (FPS)**Input:**$\;\mathrm{Point}\;\mathrm{set}\;{{\text{\{}p}_{i}\text{\}}}_{i=1,2,\text{...}N}\in {\mathbb{R}}^{N\times 5}$. **Output:** Sampled point set ${\left\{q_{i}\right\}}_{i=1,2,\text{...}N_{1}}\in {\mathbb{R}}^{N_{1}\times 5}$.  1.Select the first point in the input point cloud as the starting point and add it to the sampled point set to obtain $s=\left\{q_{0}\right\}$; 2.Calculate the distances between all points in the input point set and $q_{0}$ using Equation (3) to form an N-dimensional array D. Add the point corresponding to the maximum value in the array D to the sampled point set to obtain $s=\left\{q_{0},q_{1}\right\}$; 3.Calculate the distance from all points of point set to the newly joined points, and, for each point $p_{i}$, update D[i] if this distance is less than D[i]; 4.Select the point corresponding to the maximum value in the array D to add to the sampled point set, i.e., $s=\left\{q_{0},q_{1},q_{2}\right\}$; 5.Repeat steps 2–4 until there are $N_{1}$ points in the sampled point set.



Unlike PointNet++, we consider that the radar point cloud has higher sparsity than the laser point cloud or the point cloud generated by CAD. Therefore, we chose the nearest neighbor algorithm for grouping. Each centroid finds a set of k points nearest to it in the Euclidean space to form a group. In this way, we obtain the features of the point set group with dimension $N_{1}\times k\times c$ and $N_{1}\times k\times d_{i}$. After transforming the spatial coordinate information into local coordinate information relative to the centroid, we concatenate the two features and extract the grouped features through an MLP (LBR+LBR). Lastly, by max pooling, we obtain the abstract pattern $N_{1}\times (d_{o}+c)$ at this level. In this paper, we take $N=N_{1}/2$.

### 2.4. Scalar Attention Module

**Figure 3:**
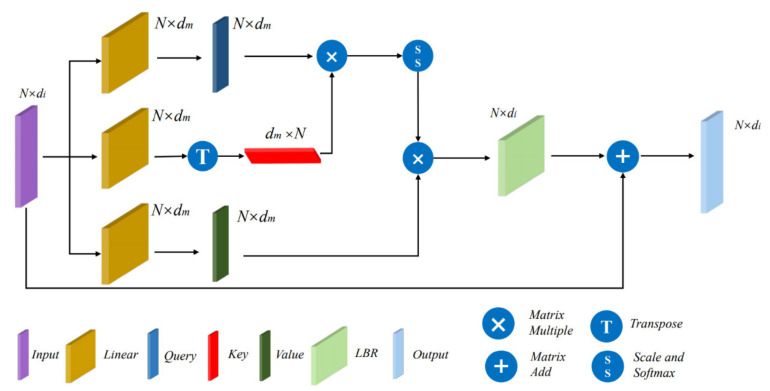
The structure of scalar attention module.

We use the self-attention module in transformer as the final global feature integration. Its essence is dot-product attention, which is also known as scalar attention [32]. The structure of the scalar attention module is shown in Figure 3.

Given the input features $F_{i}\in {\mathbb{R}}^{N\times d_{i}}$, its query, key and value matrices are obtained by three linear layers, i.e., Q, K and V, respectively.

(4) $$\begin{array}{l} \left(Q,K,V)=Linear_{1,2,3}(F_{i}\right)\\ Q,K,V\in {\mathbb{R}}^{N\times d_{m}} \end{array}$$

According to transformer, we get the attention weights $\alpha^{\prime }$ by matrix dot-product, performed to get the normalized attention weights α. Weighted with value, we get the attention feature $F_{o}{}^{\prime }$ as follows:

(5) $$\alpha^{\prime }=Q\cdot K^{T},\alpha^{\prime }\in {\mathbb{R}}^{N\times N},$$

(6) $$\alpha =softmax(\frac{\alpha^{\prime }}{\sqrt{d_{m}}}),\alpha \in {\mathbb{R}}^{N\times N},$$

(7) $$F_{o}{}^{\prime }=\alpha \cdot V,F_{o}{}^{\prime }\in {\mathbb{R}}^{N\times d_{m}}.$$

Lastly, the attention feature $F_{o}{}^{\prime }$ is connected with the input feature by a short-cut structure after LBR, and the final output $F_{o}$ is expressed as

(8) $$F_{o}=LBR(F_{o}{}^{\prime })+F_{i}.$$

### 2.5. Vector Attention Module

**Figure 4:**
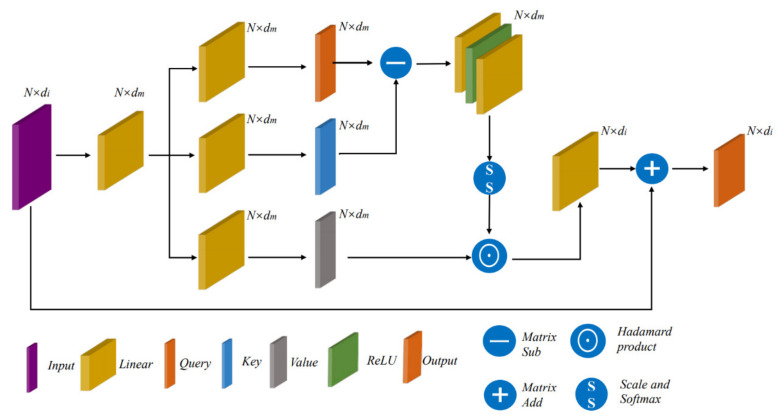
The structure of vector attention module.

Inspired by [32], we use the vector attention module in this paper. Unlike the original vector attention, we do not perform weighted accumulation in the orientation of the local footprint. Instead, we do a Hadamard product operation on value and attention, which can adjust the individual feature channels. The structure of the scalar attention module is shown in Figure 4.

We express it as

(9) $$F_{o}=\rho (\gamma (\delta (\varphi (F_{i}),\psi (F_{i}))))⊙\beta (F_{i}),$$

where $F_{i}\in {\mathbb{R}}^{N\times d_{m}}$ is the feature obtained by a linear transformation of the input, while φ and ψ are linear transformations that can be trained. Through two linear transformations, we get Q and K matrices corresponding to scalar attention, which have the same dimension, $Q\in {\mathbb{R}}^{N\times d_{m}},K\in {\mathbb{R}}^{N\times d_{m}}$. δ is the relational function; here, we use subtraction, i.e., Q−K.

To make attention more expressive, we additionally introduce the γ function, which consists of two linear layers and a ReLU activation function. ρ is the normalization function, which is normalized according to the scaled and softmax operations in the transformer to get the final attention. Here, the scaled operation is $1/\sqrt{d_{m}}$. β is also a linear transformation, which transforms the input features into the value matrix, i.e., $V\in {\mathbb{R}}^{N\times d_{m}}$. We perform a Hadamard product operation on attention and value, which multiplies each feature channel by a weighting factor.

## 3. Results

### 3.1. Dataset

#### 3.1.1. Experimental Equipment

As we know, deep learning is data-driven, and quality data are crucial for neural network training. At present, the public datasets containing radar information mainly include Nuscence [35], CRUW [36] and Oxford Radar Robotcar Dataset [37]. However, these datasets contain only 2D radar point information or radiofrequency (RF) images of the radar. The Astyx Dataset is the only autonomous driving dataset that focuses on imaging MMW radars. However, it has just over 500 frames and is very unbalanced in terms of classes, most of which are cars. To solve the above problem, we collected and created our own imaging radar classification dataset, which contains 10,000 frames and five classes, i.e., persons, cyclists, motorcyclists, cars and buses.

**Figure 5:**
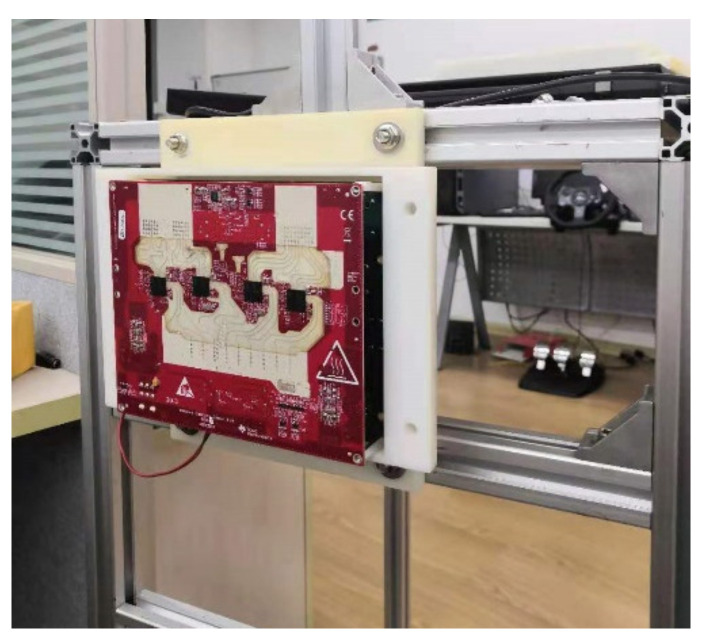
The structure of the imaging radar.

Our acquisition equipment was a TI imaging radar TIDEP01012, composed of four AWR2243 cascaded radar boards. In medium-range applications (150 m ranges), creating an MIMO antenna arrays across multiple cascaded AWR2243 devices allows us to maximize the number of active antennas, enabling substantially improved angular resolution. The structure and parameters of the imaging radar are shown in Figure 5 and Table 1, respectively.

#### 3.1.2. Radar Signal Processing

**Figure 6:**
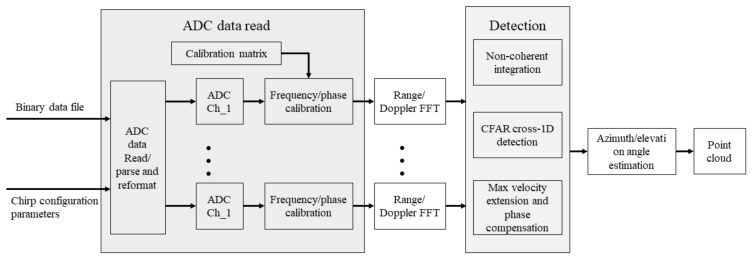
The radar signal processing flow.

Our imaging radar development board was designed in a cascade of four devices. Antenna calibration was required to prevent frequency, phase and amplitude mismatches between the master device and the remaining three slave devices caused by the differences between chips and antenna coupling, and other factors. We used the TI official calibration matrix by one-time boresight calibration. Then, we processed the data that were actually collected. The radar signal processing flow is shown in Figure 6.

Our chirp configuration parameters were set to those in MIMO mode. The ADC data were first to read and parsed, and then frequency and phase calibrations were performed. The calibrated data were subjected to range fast Fourier transform (FFT) and Doppler FFT. Since there were multiple channels, non-coherent integration needed to be done. In order to filter out noise and interference, the constant false-alarm rate (CFAR) algorithm was then performed. After performing maximum velocity extension and phase compensation, azimuth and elevation angle estimation were performed to obtain the final point cloud.

#### 3.1.3. Data Acquisition and Production

We collected data in static scenes and dynamic scenes. Static data were collected in an open experimental site. In order to fully collect the data of the object at different distances and angles, we collected data at a distance interval of 1 m and an angle interval of 45° in the range of 5–40 m from the object, which can fully represent the distribution of the object point cloud. To make the object classes more representative, we collected different types of samples for each class of objects, such as multiple persons with different shapes, and different types of bicycles, cars, etc. For dynamic data, we collected them on campus roads and experimental sites, and different objects moved at different speeds and angles.

The format of the reflected points in each frame was $p_{i}=\{r_{i},\theta_{i},\varphi_{i},v_{i},s_{i}\}$, where $r_{i}$ is the range, $\theta_{i}$ is the azimuth angle, $\varphi_{i}$ is the elevation angle, $v_{i}$ is the radial velocity, and $s_{i}$ is the signal-to-noise ratio. We used Equation (10) to convert the radar points from the original spherical coordinate system to the Cartesian coordinate system for subsequent analysis, visualization and labeling.

(10) $$\left[\begin{array}{l} x_{i}\\ y_{i}\\ z_{i} \end{array}\right]=r_{i}\left[\begin{array}{l} cos(\theta_{i})cos(\varphi_{i})\\ sin(\theta_{i})cos(\varphi_{i})\\ sin(\varphi_{i}) \end{array}\right].$$

**Figure 7:**
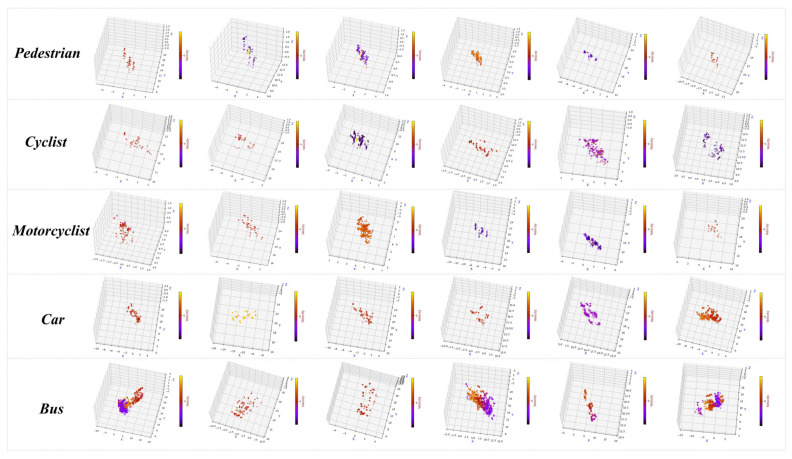
Visualization of some experimental data.

We firstly clustered the obtained point cloud for each frame to get the approximate 3D bounding box. Then, we carried out a manual correction to retain the object point cloud and labeled it with the information recorded by the camera. Our final dataset contained 10,000 frames with a mix of dynamic and static data. This is because the vehicle class contained objects in multiple states of motion on the actual road. Some experimental data are shown in Figure 7.

### 3.2. Experimental Details

We collected 10,000 frames of data, classifying road participants into five classes: person, cyclist, motorcyclist, car and bus. Our data classes were proportionally balanced, i.e., each class had 2000 frames of data. We randomly selected each class in a 7:3 distribution to yield 7000 frames of data for training and 3000 frames for testing. Each point in the object point cloud had xyz spatial information, Doppler velocity v and intensity information s. Since the proposed network needed to use this five-dimensional information, we used Z-score standardization for each frame of data and unitized it in the high-dimensional space. Suppose there are N points per frame; then, each point is described as $p_{i}=\{x_{i},y_{i},z_{i},v_{i},s_{i}\}$.

(11) $$(x_{i},y_{i},z_{i},v_{i},s_{i})=\frac{\left(x_{i},y_{i},z_{i},v_{i},s_{i}\right)}{max(\sqrt{x_{i}{}^{2}+y_{i}{}^{2}+z_{i}{}^{2}+v_{i}{}^{2}+s_{i}{}^{2}})},i=1,2,\ldots N.$$

Our network needed to input the same number of points for input embedding. Since the radar point cloud was relatively sparse, we chose 128 input points, and the effect of different input points is discussed in Section 4. If very few tiny objects at long distances had fewer than 128 points, such as distant pedestrians, we supplemented the missing points with zero elements. If there were more than 128 points, to better cover the point set, we used FPS for sampling, whose distance metric was the distance in a Euclidean space of dimension 5.

Our implementation of the Radar Transformer was based on PyTorch [38]. We chose the stochastic gradient descent (SGD) optimizer, whose momentum and weight decay were 0.9 and 0.0001, respectively. The initial learning rate was set to 0.001, and the learning rate decayed by 30% every 20 epochs. We used softmax cross-entropy as the loss function. During training, we augmented the input data with random drop points (set to 0), [−0.1, 0.1] random translation, and [0.8, 1.25] anisotropic random scaling. During testing, we did not perform data augmentation. We trained a total of 200 epochs with a batch size of 24, and all experiments were done with a GeForce GTX 1080ti.

### 3.3. Evaluation Metrics

For the object classification tasks, overall accuracy (OA), single-class accuracy and confusion matrices are commonly used quantitative evaluation metrics. The overall accuracy is the number of correct predictions for all classes as a percentage of the total number of samples, which can measure the overall performance of the model. The single-class accuracy refers to the percentage of correct predictions of each class in the total of each class, which represents the classification performance of the model for each class. Moreover, the confusion matrix counts the classes of correct and incorrect object predictions for each class, converging all of them into a table that is used to represent the confusion between different classes. We conducted several experiments and averaged them as the final result.

### 3.4. Experimental Results

We tested the performance of several popular point cloud networks such as PointNet, PointNet++, DGCNN, PCT and PointConv on our dataset and compared them with our proposed Radar Transformer. The results are shown in Table 2.

Since our dataset had fewer points per frame than datasets such as Modelnet40 [17], we made reasonable adjustments to the popular point cloud network. We mainly scaled the parameters, such as the number of input points and the number of sampling points, while the remaining parameters and training strategies were kept the same as the original paper.

From Table 2, we can see that our method achieved SOTA performance in the imaging radar classification dataset. Our overall accuracy was 94.9%, which was 1.6% higher than the second-ranked PointNet++ (MSG). Our method achieved 96.4%, 89.1%, 93.0%, 96.6% and 99.6% accuracy, respectively, for the classifications of person, cyclist, motorcyclist, car and bus. These were all the highest scores, except for bus accuracy, which was equal to PointNet++ (MSG). These findings fully demonstrate the effectiveness of our method.

By comparing the classification accuracy of each class, we found that most of the networks had a higher classification accuracy for both cars and buses. We believe that these two classes of objects can be easily distinguished in terms of both shape features and local feature abstraction. For example, both PointNet and PCT can better learn their abstract representations on global features, while PointNet++ and DGCNN continuously abstract local features and can also classify them well. For the two object classes of cyclists and motorcyclists, most networks performed poorly. For example, the classification accuracy of PointNet in these two classes was only 69.0% and 80.6%, respectively, and the other networks achieved only about 70–90%. However, the accuracy of our method for these two classes was 89.1% and 93.0%, respectively, which were both the highest values. This is because, for these two types of objects with extremely similar appearance and local information, the distribution of their features in high-dimensional space must be different. Our network fused local features and global features in high-dimensional space, which allowed us to better abstract the representation of object features.

**Figure 8:**
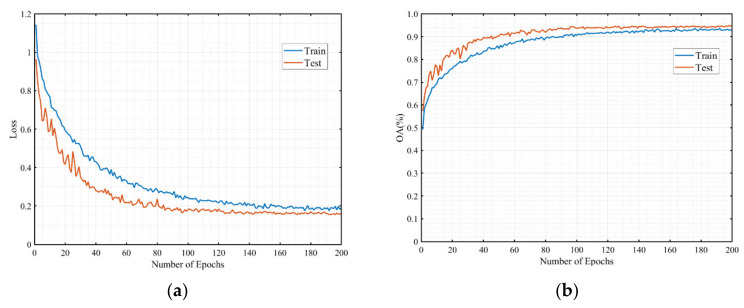
The curves of loss and overall accuracy. (**a**) Loss curve, (**b**) Accuracy curve.

Figure 8a,b show the loss curve and the overall accuracy curve, respectively. From Figure 8, we can see that the loss of training and testing decreased and accuracy increased as the number of epochs increased, and the two trends were always consistent. Moreover, our test accuracy was slightly better than the training accuracy, which indicates that our model learned the intrinsic features of the data better, had good generalization, and did not suffer from overfitting.

**Figure 9:**
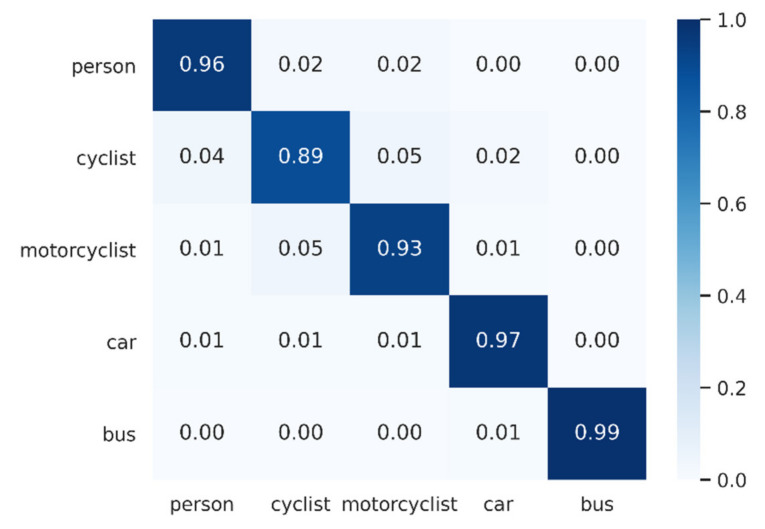
Confusion matrix of Radar Transformer with our dataset.

We plotted a confusion matrix of our model prediction results (see Figure 9). The horizontal axis represents the model’s prediction labels, while the vertical axis represents the actual labels, as well as the values representing the percentage of predictions in a particular class.

Persons, cyclists and motorcyclists are relatively small objects, and they were misclassified more severely than cars and buses. Although our model achieved relatively good results, there is still space for improvement.

**Figure 10:**
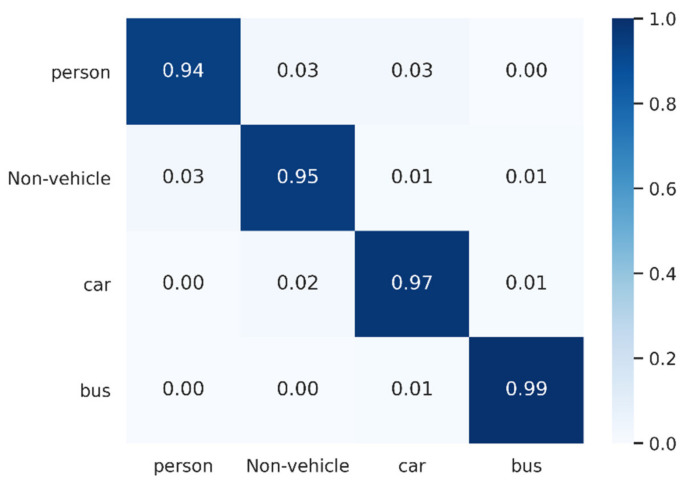
Confusion matrix of Radar Transformer with our dataset (four classes).

Furthermore, we considered the actual situation in which cyclists and motorcyclists have strong similarities. We classified them into one class, i.e., non-vehicles, to see how the model performed and the degree of confusion with other classes, as shown in Figure 10. In this case, the accuracy of non-vehicles reached 95.31%, and the degree of confusion between them and persons was reduced, with an overall accuracy of 96.42%. In practical applications, this approach makes sense.

## 4. Discussion

In this section, we explore the impact of several important parameters on network performance.

**Figure 11:**
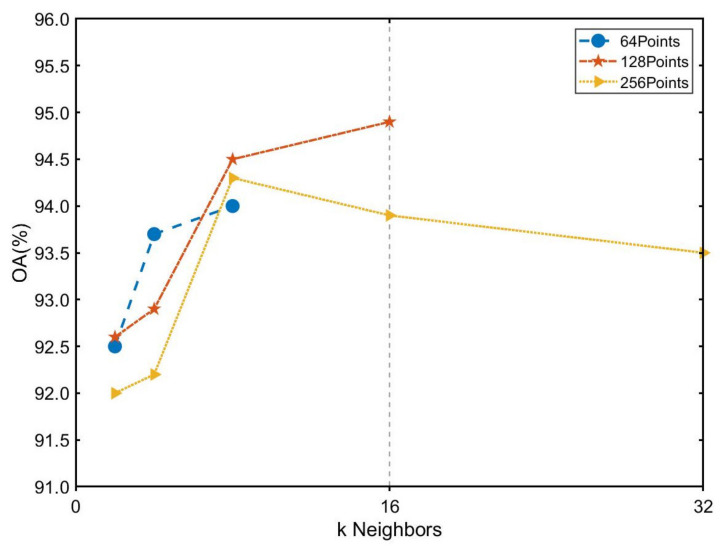
The influence of the number of input radar points and neighbors.

First of all, we consider the influence of the number of input radar points and neighbors on the network performance. The number of neighbors k represented the nearest k neighboring points of the sampled points of that hierarchical level at each extraction of our local hierarchical features. We took the number of input points as 64, 128 and 256 and the number of neighbors as 2, 4, 8, 16 and 32. However, the number of points in the last local feature layer of our network was downsampled to one-eighth of the original input, such that when the input was 64 and 128, not all neighbors could be taken. The experimental result is shown in Figure 11.

We found that our network performed best when the number of input points was 128, and the number of neighbors k was taken as 16. For input points, when the number of input points was relatively small (64 points), the network performance improvement was bottlenecked because it is challenging to represent the target information with too few points. However, when there were too many points (256 points) affected by small and medium-sized objects in the sample, many weak points were introduced, resulting in inaccurate network prediction. For the number of neighbors k, when it was small (k = 2, 4 or 8), the network was unable to obtain enough information and did not extract comprehensive local feature information during local hierarchical feature extraction. When k was too large (k = 32), this caused interference with the local features due to their possible low correlation, causing a decrease in the accuracy of the network prediction.

Furthermore, we also investigated the effect of the feature integration layer and the number of scalar attention modules on the results. Unlike vector attention, scalar attention is attention-weighted for different feature channels, which facilitates final feature learning and integration for the final deep global features. We used 0, 1, 2 or 3 scalar attention modules, and the results of the network tests are shown in Table 3.

The experimental results show that, when the number of scalar attention modules was 1, the network test results were the best, indicating that the scalar attention module can better integrate deep global features. In our imaging radar classification dataset, it showed higher performance with an overall classification accuracy of 94.9%, indicating that it is more suitable for processing sparse but high-quality radar point clouds.

In addition, we discuss below the computation time and resource consumption of our network during the training phase and evaluation phase and compare them with other methods. We counted the number of parameters, the average training time per epoch (Train_time), the video memory required during training (VRAM_train), the average inference time per batch (Infer_time) and the video memory required (VRAM_infer) during evaluation for all methods. The results are shown in Table 4.

We trained and evaluated all methods on a workstation with an Intel Xeon CPU E5-2630 v4 and a GeForce GTX 1080ti. During the evaluation phase, we had a batch size of 8. As seen in Table 4, our model had relatively lower time and video memory consumption with the highest accuracy and achieved a good trade-off.

## 5. Conclusions

In this paper, we presented a new object classification method for 4D MMW imaging radar. Influenced by transformer, our network architecture took the attention mechanism as the core and creatively fused global features with local hierarchical features in depth. We combined two attention mechanisms, scalar attention and vector attention, to effectively perform high-level feature extraction on radar point clouds. In the future, we will continue to study more effective feature extraction methods for imaging radars and apply them to new fields, such as imaging radar-based 3D detection and other tasks.

## Figures and Tables

**Table 1:** The parameters of the imaging radar.

Parameter	Value (MIMO)
Maximum range	150 m
Range resolution	60 cm
Azimuth angle resolution	1.4°
Elevation angle resolution	18°
Maximum velocity	133 kph
Velocity resolution	0.53 kph
Antennas	12 × TX, 16 × RX
Azimuth array	86 element virtual array
Elevation array	4 element virtual array

**Table 2:** Object classification results on our dataset.

Method	OA	Person	Cyclist	Motorcyclist	Car	Bus
PointNet [12]	87.0%	91.5%	69.0%	80.6%	93.3%	97.8%
PointNet++ (SSG) [21]	91.0%	93.0%	82.0%	85.1%	94.5%	98.6%
PointNet++ (MSG) [21]	93.3%	94.8%	86.1%	90.1%	95.8%	**99.5%**
DGCNN [20]	90.1%	92.3%	77.3%	88.1%	94.1%	98.5%
PCT [31]	86.4%	87.3%	76.1%	79.5%	90.6%	98.5%
NPCT [31]	87.5%	88.5%	80.3%	79.0%	91.5%	98.1%
SPCT [31]	93.0%	95.1%	83.6%	90.8%	96.1%	99.1%
PointConv [23]	89.8%	93.3%	80.1%	81.1%	95.6%	98.8%
Radar Transformer	**94.9%**	**96.4%**	**89.1%**	**93.0%**	**96.8%**	99.4%

The highest accuracy for each class are bolded to make it easy to see which method the best performance belongs to.

**Table 3:** Effect of scalar attention number.

**Scalar Attention Number**	0	1	2	3
**OA (%)**	91.5%	94.9%	91.0%	60.0%

**Table 4:** Performance analysis of the proposed method.

Method	Params (M)	Train_Time (s/epoch)	VRAM_Train (MB)	Infer_Time (ms)	VRAM_Infer (MB)
PointNet [12]	3.46	9.71	693	3.71	623
PointNet++ (SSG) [21]	1.47	19.12	683	40.38	571
PointNet++ (MSG) [21]	1.74	22.24	927	45.13	679
DGCNN [20]	1.81	4.89	807	2.80	647
PCT [31]	2.88	15.12	789	20.98	657
NPCT [31]	1.36	7.32	611	3.74	577
SPCT [31]	1.36	7.50	613	3.82	578
PointConv [23]	19.56	33.22	1835	66.88	1119
Radar Transformer	2.28	20.94	689	19.87	620

## Data Availability

The data presented in this study are available on request from the corresponding author.
